# Author Correction: Synthetic CRISPR-Cas gene activators for transcriptional reprogramming in bacteria

**DOI:** 10.1038/s41467-018-06909-4

**Published:** 2018-10-15

**Authors:** Chen Dong, Jason Fontana, Anika Patel, James M. Carothers, Jesse G. Zalatan

**Affiliations:** 1 Department of Chemistry, University of Washington, Seattle, WA 98195 USA; 2 Molecular Engineering & Sciences Institute, University of Washington, Seattle, WA 98195 USA; 3 Department of Chemical Engineering, University of Washington, Seattle, WA 98195 USA; 4 Center for Synthetic Biology, University of Washington, Seattle, WA 98195 USA

**Correction to**: *Nature Communications* 10.1038/s41467-018-04901-6; published online 27 June 2018

In the original version of the Supplementary Information file associated with this Article, the sequence ‘1x MS2 scRNA.b2’ was incorrectly given as ‘GAAGATCCGGCCTGCAGCCAGTTTTAGAGCTAGAAATAGCAAGTTAAAATAAGGCTAGTCCGTTATCAACTTGAAAAAGTGGCGCACATGAGGATCACCCATGTGCTTTTTT’ and should have read ‘GAAGATCCGGCCTGCAGCCAGTTTTAGAGCTAGAAATAGCAAGTTAAAATAAGGCTAGTCCGTTATCAACTTGAAAAAGTGGCACATGAGGATCACCCATGTGCTTTTTTT’. The error has now been fixed and the corrected version of the Supplementary Information PDF is available to download from the HTML version of the Article.

