# Supplementary material for: Synthetic CRISPR-Cas gene activators for transcriptional reprogramming in bacteria
Source: Nat Commun. 2018 Jun 27;9:2489. doi: 10.1038/s41467-018-04901-6 (PMC6021436; doi:10.1038/s41467-018-04901-6)
Supplement: Supplementary file 1 — Supplementary Information [file 41467_2018_4901_MOESM1_ESM.pdf]

SUPPLEMENTARY INFORMATION

Synthetic CRISPR-Cas Gene Activators for Transcriptional Reprogramming in Bacteria

Chen Dong, Jason Fontana, Anika Patel, James M. Carothers, & Jesse G. Zalatan*

Supplementary Figures

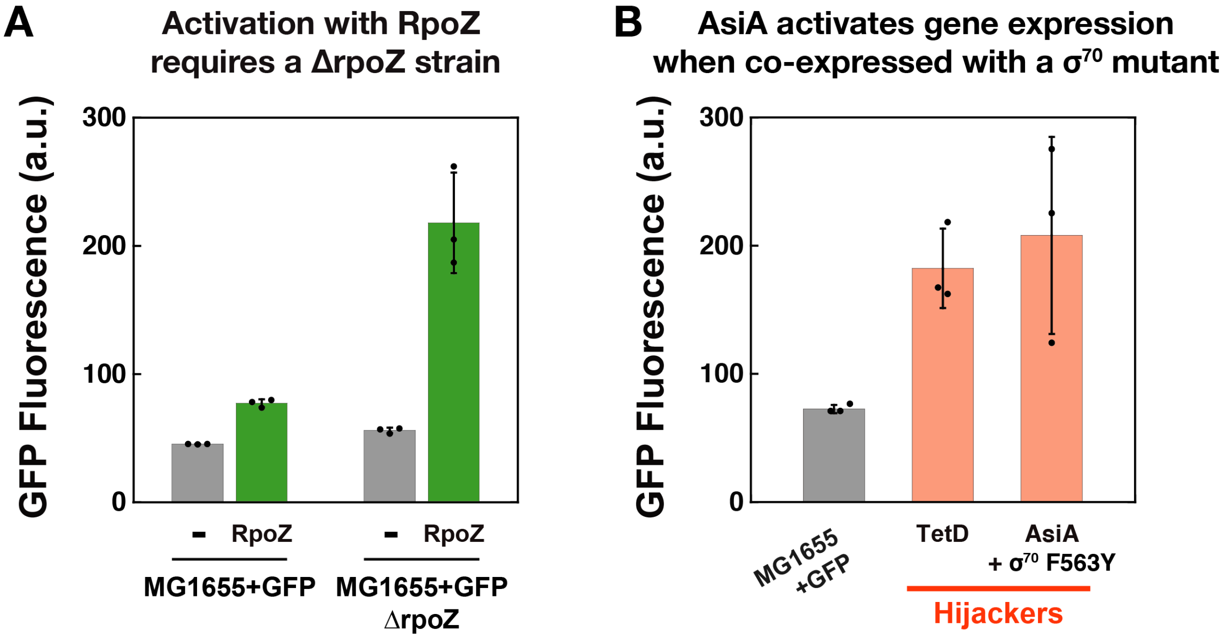

Supplementary Figure 1. Some candidate transcriptional activation domains require additional strain modifications for effective reporter gene activation.

A) CRISPRa with the MCP-RpoZ activator recruited via an scRNA is significantly increased in a ΔrpoZ host strain, consistent with that observed previously for other RpoZ fusion proteins including dCas9-RpoZ[1,2]. GFP reporter strains were transformed with dCas9 and a 1x MS2 scRNA, and either with or without the MCP-RpoZ fusion protein.

B) CRISPRa with an MCP-AsiA transcriptional activator, co-transformed with a σ^{70} F563Y mutant, produces GFP expression levels comparable to that obtained with MCP-TetD. The σ^{70} F563Y mutant prevents toxicity that occurs when AsiA is expressed alone and inhibits the activity of the endogenous σ^{70} subunit[3]. Attempts to transform MCP-AsiA into *E. coli* without co-transforming σ^{70} F563Y were unsuccessful unless a substantially weaker promoter (BBa_J23112) was used for MCP-AsiA, and no detectable GFP expression was observed with this construct (data not shown).

Values reported are GFP fluorescence levels measured by flow cytometry. Values are median ± s.d. for at least three biological replicates (specific values are indicated by black dots).

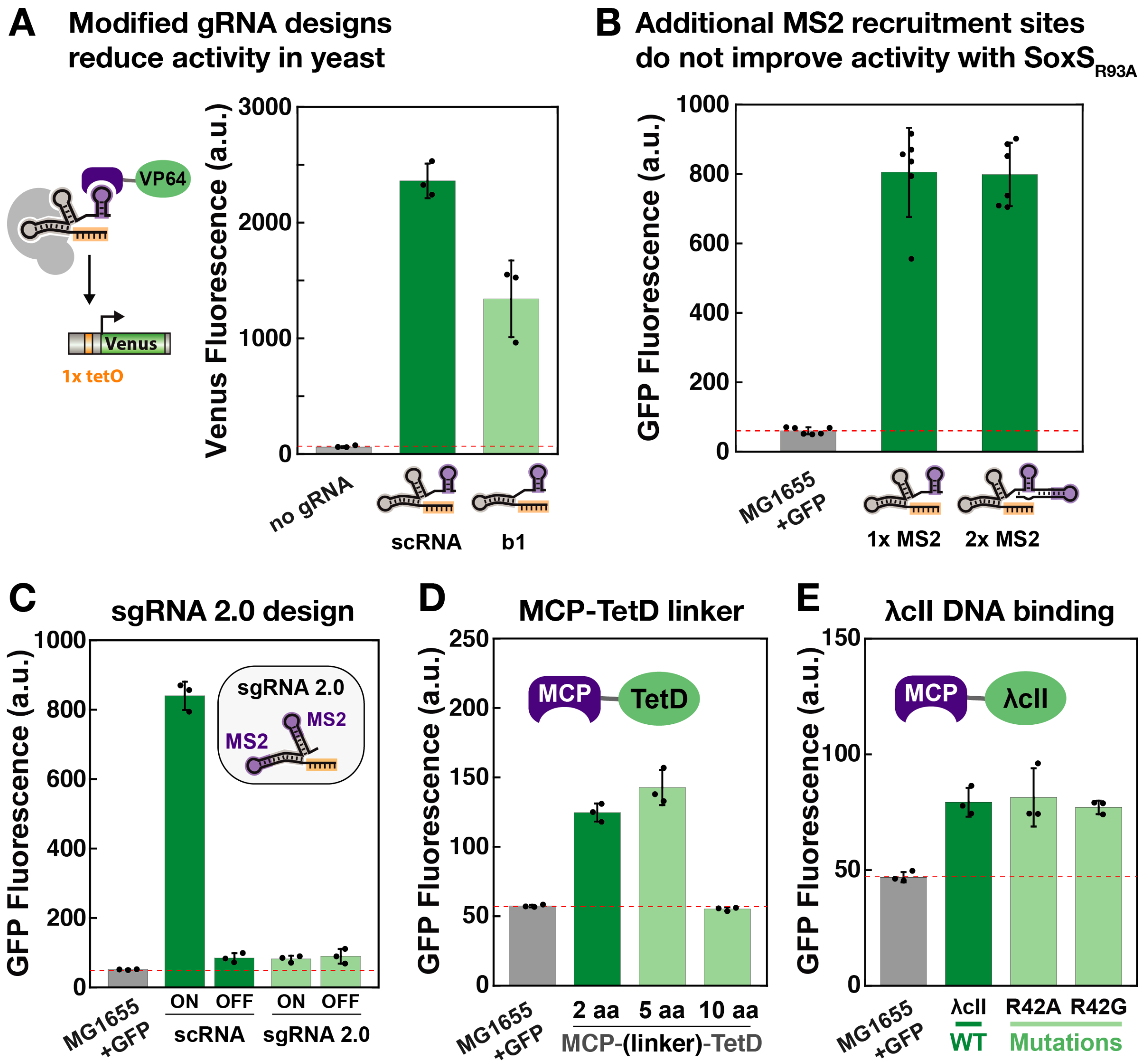

Supplementary Figure 2. Optimization of gene activation.
A) Removing the 3' terminator hairpin from a 1x MS2 scRNA decreases CRISPRa in a yeast reporter system. See Supplementary Methods for complete sequences of 1x MS2 scRNA and 1x MS2 scRNA.b1. Experiments were performed in yeast as previously described using a single TetO target site to activate a Venus fluorescent reporter gene by recruiting MCP-VP64, an activator of eukaryotic transcription[4].
B) A 2x MS2 scRNA, which increases CRISPRa in yeast and human cells relative to a 1x MS2 scRNA[4], does not improve GFP expression with CRISPRa in *E. coli*. The activator in this experiment is MCP-(5aa)-$SoxS_{R93A}$ (Fig. 3).
C) sgRNA 2.0 does not activate GFP expression in *E. coli*. The sgRNA 2.0 design has two MS2 hairpins embedded wthin internal hairpins of the sgRNA and is very effective for CRISPRa in eukaryotic cells[5]. We expressed either 1x MS2 scRNA or sgRNA 2.0 with a W108 target site (ON)

or an off-target RR2 sequence (OFF). The activator in this experiment is MCP-(5aa)-$SoxS_{R93A}$ (Fig. 3).
D) Increasing the linker length between MCP and TetD from 2 to 5 amino acids modestly increases GFP expression, while increasing the linker further to 10 amino acids decreases GFP expression to background levels. The optimized 1x MS2.b1 scRNA was used in this experiment.
E) Mutations in the MCP-λcII activator at R42, which is essential for DNA binding, retain activity when recruited via CRISPRa in *E. coli*, although the absolute level of GFP expression remains low compared to CRISPRa with MCP-SoxS. The unmodified 1x MS2 scRNA was used in this experiment. λcII R42G was previously described[6].
Values reported are Venus or GFP fluorescence levels measured by flow cytometry. Values are median ± s.d. for at least three measurements (specific values are indicated by black dots).

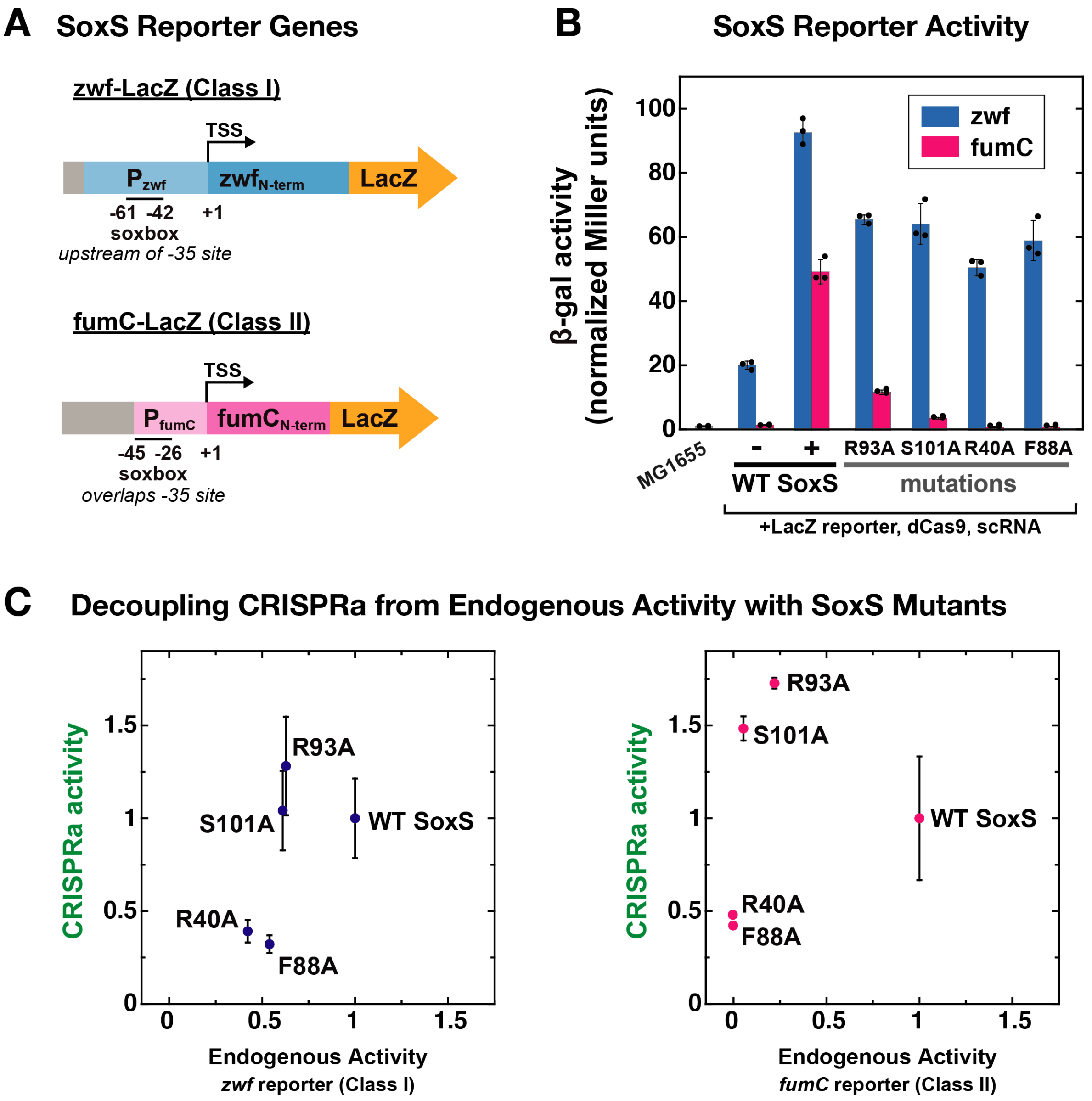

Supplementary Figure 3. Mutations in SoxS reduce activity at endogenous SoxS promoters.

A) Plasmids containing zwf-LacZ and fumC-LacZ fusion constructs were used as reporters of endogenous SoxS transcriptional activity[7]. The *zwf* promoter is representative of SoxS class I promoters in which the SoxS target site (soxbox) is upstream of the -35 site. The *fumC* promoter is representative of SoxS class II promoters in which the soxbox overlaps the -35 site. Complete sequences of the reporter constructs are included in Supplementary Methods.

B) LacZ (β-Gal) activity was measured in *E. coli* strain CD06 (Supplementary Table 1), an MG1655 strain modified to include a GFP reporter for CRISPRa activity. This strain was further transformed with a plasmid containing either the zwf-LacZ or fumC-LacZ reporter, along with a plasmid with the CRISPRa system components dCas9, a 1x MS2 scRNA.b1, and an MCP-SoxS fusion protein. Point mutants of SoxS that disrupt the DNA binding interface lead to reduced LacZ activity, and the results are consistent with previously reported activity trends for these mutants[7]. LacZ activity was measured as previously described[8], and values are reported in Miller units

normalized to the value obtained in the MG1655 parent strain with no reporter plasmids. Values are median ± s.d. for three measurements (specific values are indicated by black dots).
C) Plots of CRISPRa activity vs endogenous activity for wild type (wt) and mutant SoxS proteins indicate that transcriptional activation can be decoupled from binding to endogenous targets. In the main text Fig. 3D, CRISPRa activity values were measured in strains that did not contain LacZ reporters (Fig. 3C). The CRISPRa activity values plotted here were obtained in the same strains used to measure LacZ activity. Similar trends were observed in both cases. CRISPRa activity values were normalized to the value obtained for wt SoxS. Endogenous activity values are LacZ activity (Supplementary Fig. 3B), corrected for background reporter activity in the absence of SoxS and normalized to the value obtained for wt SoxS. Values are median ± s.d. for at least three measurements.

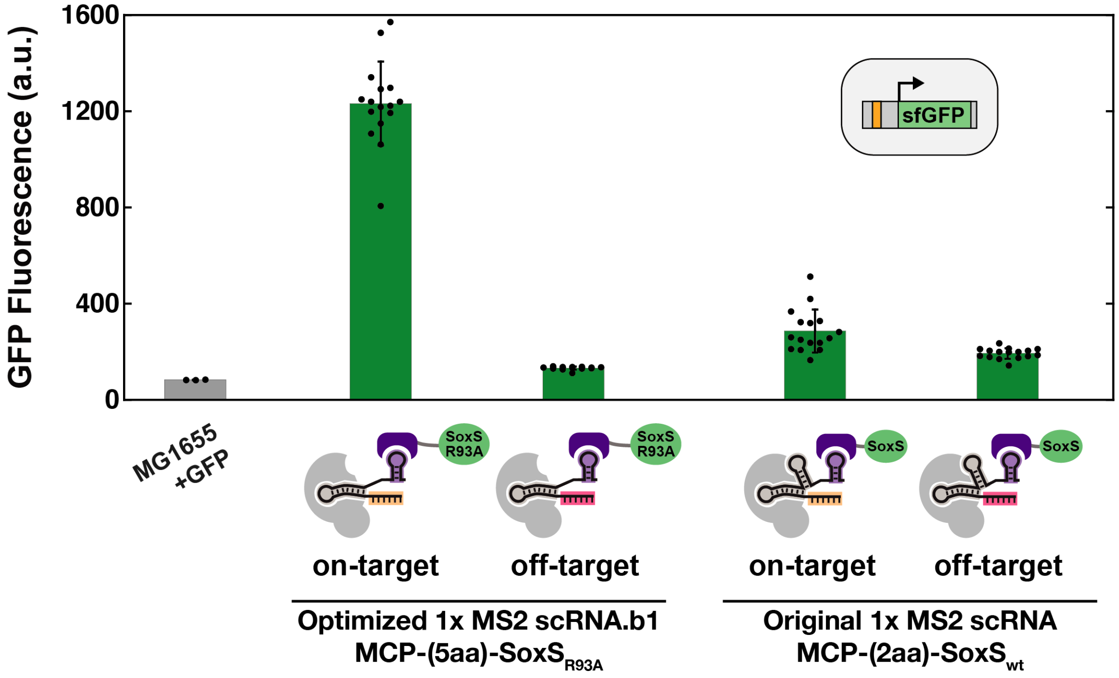

Supplementary Figure 4. An optimized CRISPRa system improves activity.
The optimized CRISPRa system with 1x MS2 scRNA.b1 and MCP-(5aa)-SoxS$_{R93A}$ produces significantly more on-target GFP expression and less off-target GFP expression than the original system with 1x MS2 scRNA with MCP-(2aa)-SoxS. The on-target site is W108 and the off target site is RR2 (Supplementary Table 2). Values reported are GFP fluorescence levels measured by flow cytometry. Values are median ± s.d. for at least three biological replicates (specific values are indicated by black dots).

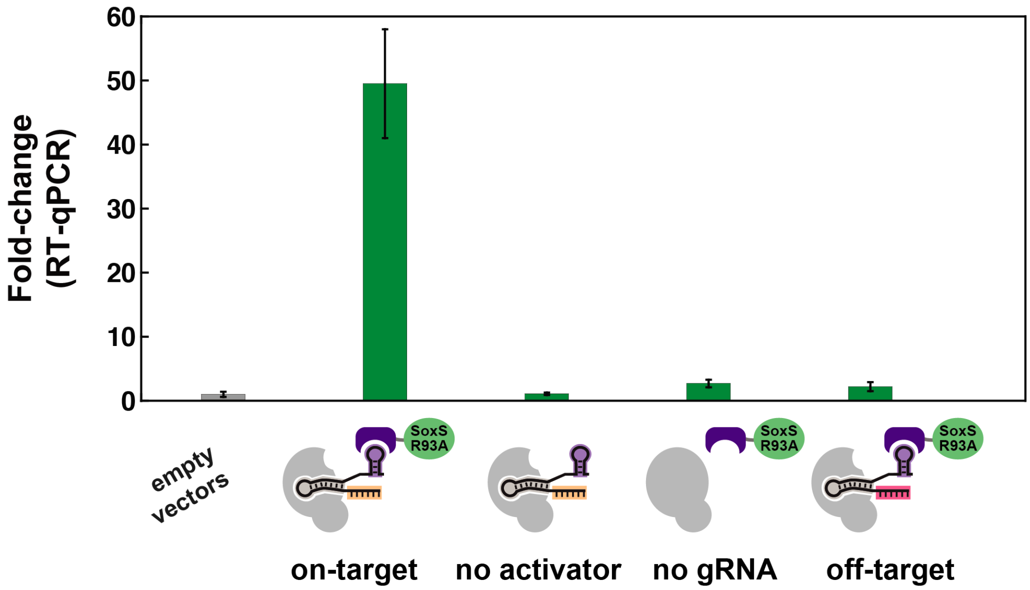

Supplementary Figure 5. CRISPRa significantly increases GFP mRNA levels.
GFP expression with a fully optimized scRNA.b1, MCP-(5aa)-$SoxS_{R93A}$ CRISPRa system increases 50-fold relative to an empty vector control, as measured by RT-qPCR. Negative controls without MCP-(5aa)-$SoxS_{R93A}$ (no activator) or scRNA.b1 produce 1.1-fold and 2.7-fold changes in GFP mRNA levels, respectively, relative to the empty vector control. When an off-target scRNA is expressed (RR2, Supplementary Table 2), there is a 2.2-fold change in GFP mRNA relative to the empty vector control. For comparison to the 50-fold increase in GFP mRNA levels with CRISPRa, we observe a 30-fold increase in GFP fluorescence in the same strain (Fig. 3E). The fluorescence change may be an underestimate of the true change in protein levels, as there is a significant autofluorescence background (see for example the observed GFP levels in the MG1655 parental strain versus MG1655+GFP in Fig. 2A). Expression levels from RT-qPCR were calculated from at least three replicates using the $\Delta\Delta C_T$ method[9] with three independent measurements each of the target gene (GFP) and reference gene (16S rRNA). Error bars represent s.e.m. Because the mean and error of fold changes in the $\Delta\Delta C_T$ method are calculated from the average and error of the individual C_T values, individual data points are not plotted.

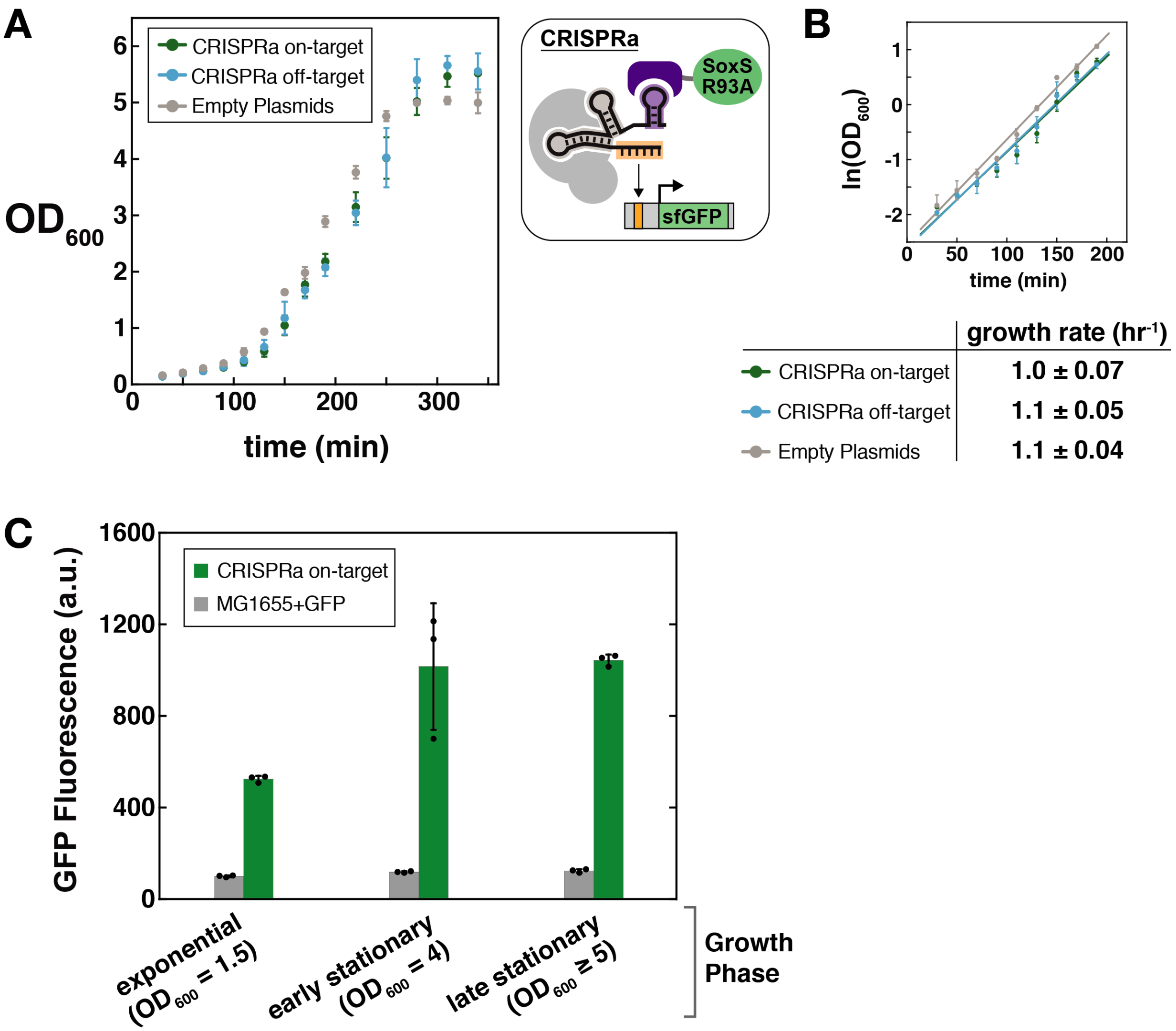

Supplementary Figure 6. Relationship between cell growth and CRISPRa.

A) The CRISPR-Cas system does not introduce a significant growth burden. Cell growth (measured by OD_{600}) versus time for *E. coli* cells transformed with empty plasmids or with CRISPRa system components, using 1x MS2 scRNAs with either on or off-target sequences. The growth curves are similar in all cases. Experiments were performed in biological triplicate, and error bars represent the standard deviation from at least three measurements.

B) Quantification of growth rates using exponential phase data only (up to 200 minutes). The slope of the plot of $\ln(OD_{600})$ versus time gives the growth rate. The observed growth rates are identical within error.

C) CRISPRa-mediated GFP expression increases as cells enter stationary phase, although gene expression is still detectable in exponential phase. Values reported are GFP fluorescence levels measured by flow cytometry. Values are median ± s.d. for at least three biological replicates (specific values are indicated by black dots).

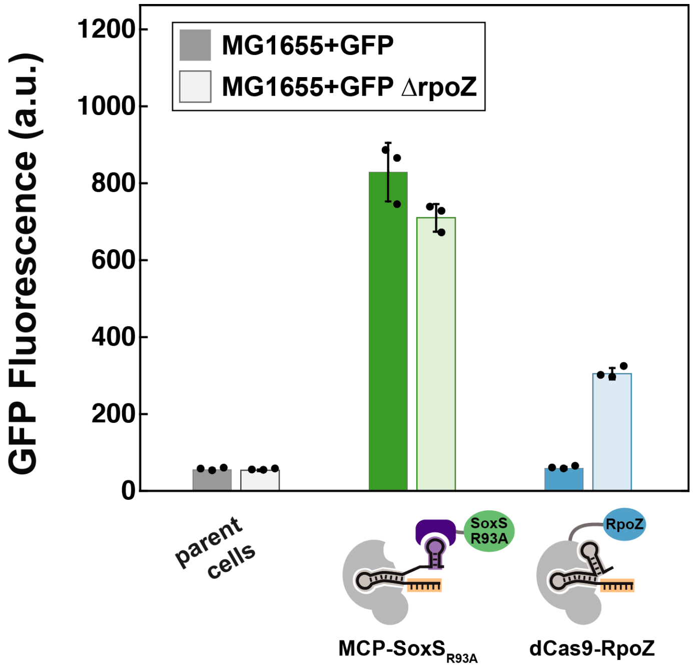

Supplementary Figure 7. CRISPRa with fully optimized MCP-SoxS$_{R93A}$ is more effective than dCas9-RpoZ, a previously-described bacterial CRISPRa system[1]. MCP-SoxS$_{R93A}$ activates gene expression in an MG1655 strain, where dCas9-RpoZ is ineffective. In a Δ*rpoZ* strain, where dCas9-RpoZ is effective, the SoxS-based system outperforms dCas9-RpoZ by >2-fold. Values reported are GFP fluorescence levels measured by flow cytometry. Values are median ± s.d. for at least three measurements (specific values are indicated by black dots).

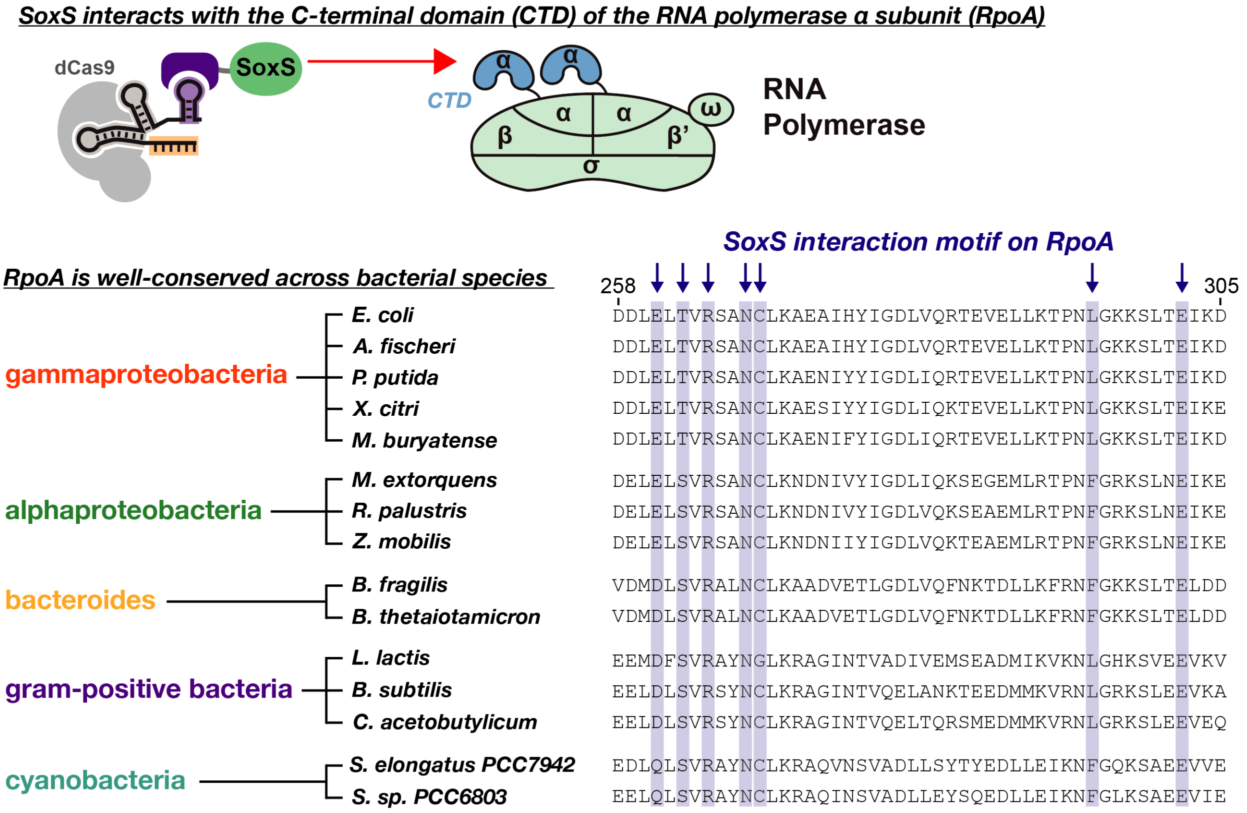

Supplementary Figure 8. The SoxS recruitment site on RNA polymerase is highly conserved. *E. coli* SoxS interacts with the C-terminal domain of RpoA, the polymerase α-subunit (αCTD), and specific amino acids on RpoA important for interacting with SoxS have been identified[10]. These amino acids in RpoA are largely conserved across a broad range of bacterial species. The observed conservation suggests that *E. coli* SoxS may be able to recruit RNA polymerase in other bacteria in addition to *E. coli*. Complete RpoA sequences were aligned using Clustal Omega[11], and the sequences corresponding *E. coli* residues 258-305 are shown.

At least seven residues have been reported to affect the *E. coli* SoxS-αCTD interaction upon alanine substitution[10]. Alanine substitutions at E261, T263, and R265 have significant detrimental effects on the SoxS-αCTD interaction. R265 is absolutely conserved among the species examined here, and E261 and T263 are largely conserved with a few homologous substitutions (i.e. E->D/Q or T->S). Alanine substitutions at L295 and E302 have modest detrimental effects. L295 is largely conserved with some homologous substitutions (L->F), and E302 is absolutely conserved. Alanine substitutions at N268 and C269 have modest beneficial effects on the SoxS-αCTD interaction; both residues are absolutely conserved except for a C->G substitution in *L. lactis*.

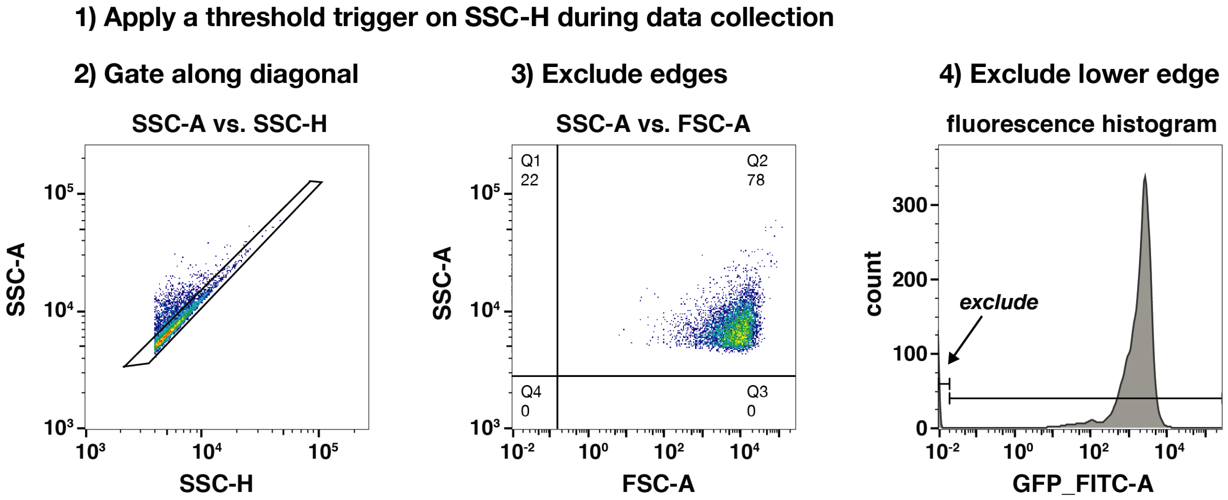

Supplementary Figure 9. Flow cytometry gating strategy. To select for single bacterial cells, we (1) applied a side scatter threshold trigger (SSC-H) during data collection. We then gated for single cells by (2) selecting events along the diagonal of the SSC-H vs. SSC-A plot[12], (3) excluding events that appeared on the edges of the SSC-A vs. FSC-A plot, and (4) excluding events that appeared on the edges of the fluorescence histogram.

Supplementary Tables

Supplementary Table 1. *E. coli* Strains

Strain	Description	Genotype
MG1655	parent *E. coli* strain	F- λ- ilvG- rfb-50 rph-1
CD03	MG1655/ΔrpoZ	MG1655 ΔrpoZ
CD06	MG1655/sfGFP (weak promoter)	MG1655 *W1-BBa_J23117-sfGFP KanR::nfsA*
CD08	MG1655/sfGFP (weak promoter)/ΔrpoZ	MG1655 *W1-BBa_J23117-sfGFP KanR::nfsA ΔrpoZ*
CD10	MG1655/sfGFP (weak promoter)/ mRFP1 (strong promoter	MG1655 *W1-BBa_J23117-sfGFP::nfsA, BBa_J23119-mRFP1 KanR::rbsAR*

Supplementary Table 2. gRNA Target Sites

**sgRNA target**	**DNA Sequence**	**Target Strand**[c]	**Distance to TSS**[d]
W108[a]	GAAGATCCGGCCTGCAGCCA	NT	91
RFP R2[b]	TGGAACCGTACTGGAACTGC	NT	-215
J101	TGGGTTCCACCGGATACCTC	T	40
J103	AGGCGTCCTTTGGGTTCCAC	T	50
J105	CGGTTACCAAAGGCGTCCTT	T	60
J107	CGGTGTCCTGCGGTTACCAA	T	70
J109	AGGTATCCTGCGGTGTCCTG	T	80
J111	GGGCGACCTCAGGTATCCTG	T	90
J113	GGGCCACCACGGGCGACCTC	T	100
J115	TGGTGACCATGGGCCACCAC	T	110
J117	GGGTGACCTATGGTGACCAT	T	120
J119	TGGTTGCCAAGGGTGACCTA	T	130
J121	AGGACACCTTTGGTTGCCAA	T	140
J102	AGGTATCCGGTGGAACCCAA	NT	61
J104	TGGAACCCAAAGGACGCCTT	NT	71
J106	AGGACGCCTTTGGTAACCGC	NT	81
J108	TGGTAACCGCAGGACACCGC	NT	91
J110	AGGACACCGCAGGATACCTG	NT	101
J112	AGGATACCTGAGGTCGCCCG	NT	111
J114	AGGTCGCCCGTGGTGGCCCA	NT	121
J116	TGGTGGCCCATGGTCACCAT	NT	131
J118	TGGTCACCATAGGTCACCCT	NT	141
J120	AGGTCACCCTTGGCAACCAA	NT	151

[a] The W108 site for GFP activation was described previously using a 30 base gRNA targeting site (i.e. spacer sequence)[1]. We use a 20 base gRNA targeting site for the same site (i.e. the same PAM site).
[b] The RFP R2 site for RFP repression by CRISPRi was described previously[13].
[c] Template strand (T) or non-template strand (NT).
[d] Distance to TSS is the distance from the 3' end (PAM proximal) of the guide target site to the transcription start site. For synthetic promoters driven by BBa_J23117 or BBa_J23119 (http://parts.igem.org), the TSS is immediately downstream of the BBa sequence (see complete maps below).

Supplementary Table 3. *E. coli* Expression Plasmids[a]

Plasmid	Marker	origin	Promoter	Gene	Terminator
pCD067	*1) AmpR*<br>*2) KanR*	*R6K*	*BBa_J23117*	sfGFP	*BBa_B0015*
pJF023	*1) AmpR*<br>*2) KanR*	*R6K*	*BBa_J23119*	mRFP1	*BBa_B0015*
p_gRNA_bact_RR2[b]	*AmpR*	*ColE1*	*BBa_J23119*	sgRNA (RR2)	*TrrnB*
pCD005	*AmpR*	*ColE1*	*BBa_J23119*	1x MS2 scRNA<br>(W108 target)	*TrrnB*
pCD006	*AmpR*	*ColE1*	*BBa_J23119*	2x MS2 scRNA<br>(W108 target)	*TrrnB*
pCD015	*AmpR*	*ColE1*	*BBa_J23119*	sgRNA (W108 target)	*TrrnB*
pCD061	*AmpR*	*ColE1*	*BBa_J23119*	1x MS2 scRNA.b1<br>(W108 target)	*TrrnB*
pJF67-2	*AmpR*	*ColE1*	*BBa_J23119*	1x MS2 scRNA.b2<br>(W108 target)	*TrrnB*
pCD034	*AmpR*	*ColE1*	*BBa_J23119*	1x MS2 scRNA<br>(RR2)	*TrrnB*
pCD315	*AmpR*	*ColE1*	*pBAD*	1x MS2 scRNA.b1<br>(W108 target)	*TrrnB*
pCD325	*AmpR*	*ColE1*	*pBAD*	sgRNA (RR2)	*TrrnB*
pCD326	*AmpR*	*ColE1*	*1) pBAD*<br>*2) pBAD*	1) 1x MS2 scRNA.b1<br>(W108 target)<br>2) sgRNA (RR2)	*1) TrrnB*<br>*2) TrrnB*
pCD372	*AmpR*	*ColE1*	*1) BBa_J23119*<br>*2) BBa_J23119*	1) 1x MS2 scRNA.b1<br>(W108 target)<br>2) sgRNA (RR2)	*1) TrrnB*<br>*2) TrrnB*
pCD403	*AmpR*	*ColE1*	*BBa_J23119*	1x MS2 scRNA.b1<br>(RR2)	*TrrnB*
pCD017	*CmR*	*p15A*	*Sp.pCas9*	dCas9	*BBa_B0015*
pCD018	*CmR*	*p15A*	*1) Sp.pCas9*<br>*2) BBa_J23107*	1) dCas9<br>2) MCP-rpoZ	*1) BBa_B0015*<br>*2) BBa_B1002*
pCD037	*CmR*	*p15A*	*1) Sp.pCas9*<br>*2) BBa_J23107*	1) dCas9<br>2) αNTD-MCP	*1) BBa_B0015*<br>*2) BBa_B1002*
pCD064	*CmR*	*p15A*	*1) Sp.pCas9*<br>*2) BBa_J23107*	1) dCas9<br>2) MCP-rpoD	*1) BBa_B0015*<br>*2) BBa_B1002*
pCD087	*CmR*	*p15A*	*1) Sp.pCas9*<br>*2) BBa_J23107*	1) dCas9<br>2) MCP-N4SSB (Y75A)	*1) BBa_B0015*<br>*2) BBa_B1002*
pCD123	*CmR*	*p15A*	*1) Sp.pCas9*<br>*2) BBa_J23107*	1) dCas9<br>2) MCP-(2aa)-SoxS (wt)	*1) BBa_B0015*<br>*2) BBa_B1002*
pCD134	*CmR*	*p15A*	*1) Sp.pCas9*<br>*2) BBa_J23107*	1) dCas9<br>2) MCP-(2aa)-MarA	*1) BBa_B0015*<br>*2) BBa_B1002*
pCD141	*CmR*	*p15A*	*1) Sp.pCas9*<br>*2) BBa_J23107*	1) dCas9<br>2) MCP-(2aa)-Rob	*1) BBa_B0015*<br>*2) BBa_B1002*
pCD146	*CmR*	*p15A*	*1) Sp.pCas9*<br>*2) BBa_J23107*	1) dCas9<br>2) MCP-(2aa)-TetD	*1) BBa_B0015*<br>*2) BBa_B1002*
pCD151	*CmR*	*p15A*	*1) Sp.pCas9*<br>*2) BBa_J23107*	1) dCas9<br>2) MCP-(2aa)-λcII	*1) BBa_B0015*<br>*2) BBa_B1002*

pCD175	*CmR*	*p15A*	*1) Sp.pCas9*<br>*2) BBa_J23107*<br>*3) BBa_J23107*	1) dCas9<br>2) MCP-AsiA<br>3) rpoD (F563Y)	*1) BBa_B0015*<br>*2) BBa_B1002*<br>*3) BBa_B1002*
pCD226	*CmR*	*p15A*	*1) Sp.pCas9*<br>*2) BBa_J23107*<br>*3) BBa_J23107*	1) dCas9<br>2) MCP-GP33	*1) BBa_B0015*<br>*2) BBa_B1002*
pCD351	*CmR*	*p15A*	*1) Sp.pCas9*<br>*2) BBa_J23107*	1) dCas9<br>2) MCP-CAP	*1) BBa_B0015*<br>*2) BBa_B1002*
pCD156	*CmR*	*p15A*	*1) Sp.pCas9*<br>*2) BBa_J23107*	1) dCas9<br>2) MCP-(5aa)-SoxS (wt)	*1) BBa_B0015*<br>*2) BBa_B1002*
pCD157	*CmR*	*p15A*	*1) Sp.pCas9*<br>*2) BBa_J23107*	1) dCas9<br>2) MCP-(10aa)-SoxS (wt)	*1) BBa_B0015*<br>*2) BBa_B1002*
pCD185	*CmR*	*p15A*	*1) Sp.pCas9*<br>*2) BBa_J23107*	1) dCas9<br>2) MCP-(10aa)-SoxS (R93A)	*1) BBa_B0015*<br>*2) BBa_B1002*
pCD186	*CmR*	*p15A*	*1) Sp.pCas9*<br>*2) BBa_J23107*	1) dCas9<br>2) MCP-(5aa)-SoxS (R93A)	*1) BBa_B0015*<br>*2) BBa_B1002*
pCD404	*CmR*	*p15A*	*1) Sp.pCas9*<br>*2) BBa_J23107*	1) dCas9<br>2) MCP-(5aa)-SoxS (F88A)	*1) BBa_B0015*<br>*2) BBa_B1002*
pCD406	*CmR*	*p15A*	*1) Sp.pCas9*<br>*2) BBa_J23107*	1) dCas9<br>2) MCP-(5aa)-SoxS (R40A)	*1) BBa_B0015*<br>*2) BBa_B1002*
pCD408	*CmR*	*p15A*	*1) Sp.pCas9*<br>*2) BBa_J23107*	1) dCas9<br>2) MCP-(5aa)-SoxS (S101A)	*1) BBa_B0015*<br>*2) BBa_B1002*
pJF093	*CmR*	*p15A*	*1) Sp.pCas9*<br>*2) TetR-pTet*	1) dCas9<br>2) MCP-(5aa)-SoxS (R93A)	*1) BBa_B0015*<br>*2) BBa_B1002*
pJF094	*CmR*	*p15A*	*1) TetR-pTet*<br>*2) Bba_J23107*	1) dCas9<br>2) MCP-(5aa)-SoxS (R93A)	*1) BBa_B0015*<br>*2) BBa_B1002*
pJF104B	*CmR*	*p15A*	*1) TetR-pTet*<br>*2) TetR-pTet*	1) dCas9<br>2) MCP-(5aa)-SoxS (R93A)	*1) BBa_B0015*<br>*2) BBa_B1002*
pJF121	*AmpR*	*ColE1*	*TetR-pTet*	1x MS2 scRNA<br>(W108 target)	*TrrnB*
pCD227	*CmR*	*p15A*	*1) pBAD*<br>*2) BBa_J23107*<br>*3) araC*	1) dCas9<br>2) MCP-(5aa)-SoxS (R93A)<br>3) AraC	*1) BBa_B0015*<br>*2) BBa_B1002*<br>*3) N/A*
pJF077.1~21[c]	*CmR*	*p15A*	*1) Sp.pCas9*<br>*2) BBa_J23107*<br>*3) BBa_J23119*	1) dCas9<br>2) MCP-(5aa)-SoxS (R93A)<br>3) 1x MS2 scRNA.b2<br>(J101~121 targets)	*1) BBa_B0015*<br>*2) BBa_B1002*<br>*3) TrrnB*
pCD294.1~21	*CmR*	*p15A*	*1) Sp.pCas9*<br>*2) BBa_J23107*<br>*3) BBa_J23119*	1) dCas9<br>2) PCP-(5aa)-SoxS (R93A)<br>3) 1x PP7 scRNA.b1<br>(J101~121 targets)	*1) BBa_B0015*<br>*2) BBa_B1002*<br>*3) TrrnB*
pCD296.1~21	*CmR*	*p15A*	*1) Sp.pCas9*<br>*2) BBa_J23107*<br>*3) BBa_J23119*	1) dCas9<br>2) MCP-(5aa)-TetD<br>3) 1x MS2 scRNA.b2<br>(J101~121 targets)	*1) BBa_B0015*<br>*2) BBa_B1002*<br>*3) TrrnB*
pCD297.1~21	*CmR*	*p15A*	*1) Sp.pCas9*<br>*2) BBa_J23107*<br>*3) BBa_J23119*	1) dCas9<br>2) αNTD-MCP<br>3) 1x MS2 scRNA.b2<br>(J101~121 targets)	*1) BBa_B0015*<br>*2) BBa_B1002*<br>*3) TrrnB*
pCD298.1~21	*CmR*	*p15A*	*1) Sp.pCas9*<br>*2) BBa_J23107*<br>*3) BBa_J23119*	1) dCas9<br>2) MCP-(2aa)-λcII (R42A)<br>3) 1x MS2 scRNA.b2<br>(J101~121 targets)	*1) BBa_B0015*<br>*2) BBa_B1002*<br>*3) TrrnB*

pCD416.1~21	CmR	p15A	*1) Sp.pCas9<br>2) BBa_J23107<br>3) BBa_J23119*	1) dCas9<br>2) MCP-(2aa)-SoxS (R93A)<br>3) 1x MS2 scRNA.b2<br>(J101~121 targets)	*1) BBa_B0015<br>2) BBa_B1002<br>3) TrrnB*
pCD417.1~21	CmR	p15A	*1) Sp.pCas9<br>2) BBa_J23107<br>3) BBa_J23119*	1) dCas9<br>2) MCP-(10aa)-SoxS (R93A)<br>3) 1x MS2 scRNA.b2<br>(J101~121 targets)	*1) BBa_B0015<br>2) BBa_B1002<br>3) TrrnB*
pCD441.1~21	CmR	p15A	*1) Sp.pCas9<br>2) BBa_J23107<br>3) BBa_J23119*	1) dCas9<br>2) MCP-(20aa)-SoxS (R93A)<br>3) 1x MS2 scRNA.b2<br>(J101~121 targets)	*1) BBa_B0015<br>2) BBa_B1002<br>3) TrrnB*
pCD290	*CmR*	*p15A*	*1) Sp.pCas9<br>2) BBa_J23107<br>3) BBa_J23119*	1) dCas9<br>2) MCP-(5aa)-SoxS (R93A)<br>3) 1x MS2 scRNA.b2<br>(J106 target)	*1) BBa_B0015<br>2) BBa_B1002<br>3) TrrnB*
pCD477	*CmR*	*p15A*	*1) Sp.pCas9<br>2) BBa_J23107<br>3) BBa_J23119*	1) dCas9<br>2) MCP-(5aa)-SoxS (wt)<br>3) 1x MS2 scRNA.b1 (W108)	*1) BBa_B0015<br>2) BBa_B1002<br>3) TrrnB*
pCD478	*CmR*	*p15A*	*1) Sp.pCas9<br>2) BBa_J23107<br>3) BBa_J23119*	1) dCas9<br>2) MCP-(5aa)-SoxS (R93A)<br>3) 1x MS2 scRNA.b1 (W108)	*1) BBa_B0015<br>2) BBa_B1002<br>3) TrrnB*
pCD479	*CmR*	*p15A*	*1) Sp.pCas9<br>2) BBa_J23107<br>3) BBa_J23119*	1) dCas9<br>2) MCP-(5aa)-SoxS (S101A)<br>3) 1x MS2 scRNA.b1 (W108)	*1) BBa_B0015<br>2) BBa_B1002<br>3) TrrnB*
pCD481	*CmR*	*p15A*	*1) Sp.pCas9<br>2) BBa_J23107<br>3) BBa_J23119*	1) dCas9<br>2) MCP-(5aa)-SoxS (F88A)<br>3) 1x MS2 scRNA.b1 (W108)	*1) BBa_B0015<br>2) BBa_B1002<br>3) TrrnB*
pCD482	*CmR*	*p15A*	*1) Sp.pCas9<br>2) BBa_J23107<br>3) BBa_J23119*	1) dCas9<br>2) MCP-(5aa)-SoxS (R40A)<br>3) 1x MS2 scRNA.b1 (W108)	*1) BBa_B0015<br>2) BBa_B1002<br>3) TrrnB*
pJF076	*AmpR*	*pSC101*	*J1_BBa_J23117*	mRFP1	*BBa_B0015*
pCD469	*AmpR*	*pSC101*	*zwf*	$zwf_{N\text{-term}}$-LacZ	*BBa_B0015*
pCD470	*AmpR*	*pSC101*	*fumC*	$fumC_{N\text{-term}}$-LacZ	*BBa_B0015*
pCD355	*AmpR*	*pSC101*	*J1_BBa_J23117*	*Z. mobilis* pdc/adhB<br>(single operon)	*BBa_B0015*
pJF009[d]	*AmpR*	*ColE1*	*n/a*	*n/a*	*n/a*
pJF043 [d]	*CmR*	*p15A*	*n/a*	*n/a*	*n/a*

[a] BBa sequences are from the Repository of Standard Biological Parts (http://parts.igem.org). dCas9 is the catalytically inactive form of *S. pyogenes* Cas9[13]. Sp.pCas9 is the endogenous Cas9 promoter from *S. pyogenes*. pBAD is an arabinose-inducible promoter, described previously[14]. Complete sequences of the GFP, RFP, and LacZ reporter constructs are included in the Supplementary Experimental Procedures.

[b] The plasmid with the RFP R2 gRNA for RFP repression was described previously[13].

[c] Plasmid designations such as pJF077.1~21 indicate a set of plasmids (pJF077.1, pJF077.2…) where the final number corresponds to guide RNA target sites (J101, J102…, Supplementary Table 2) used for the J1 mRFP1 reporter (Fig. 4).

[d] pJF009 and pJF043 are empty vector control plasmids with expression cassettes removed from p_gRNA_bact_RR2 and pCD017, respectively. These control plasmids were used in RT-qPCR experiments (Supplementary Fig. 5) and growth burden experiments (Supplementary Fig. 6).

Supplementary Table 4. Primer Sequences for RT-qPCR

Primer	Sequence
qCD010_sfGFP_f	GAGGGTGAAGGTGATGCTACAA
qCD011_sfGFP_r	GGTCAGAGTAGTGACAAGTGTTGG
qCD012_16S_f	AAAGTTAATACCTTTGCTCATTGACGTT
qCD013_16S_r	GACTACCAGGGTATCTAATCCTGTTT

Supplementary Methods

Scaffold RNA (scRNA) Sequence Designs

gRNA and 1x MS2 scRNA sequences with RNA recruitment hairpins were initially constructed following previous designs[4,13]. 1x MS2 scRNA.b1 removes the final tracr RNA terminator hairpin, based on secondary structure predictions of the hairpin structure[15]. 1x MS2 scRNA.b2 removes the final tracr RNA terminator hairpin, based on the x-ray crystal structure of gRNA in complex with Cas9 and a DNA target[16]. Compared to scRNA.b1, the scRNA.b2 design includes one additional G from the tracr sequence and removes the two base GC linker to the MS2 hairpin.

Parent sgRNA
GAAGATCCGGCCTGCAGCCAGTTTTAGAGCTAGAAATAGCAAGTTAAAATAAGGCTAGTCCGTTATCA
ACTTGAAAAAGTGGCACCGAGTCGGTGCTTTTTTT

1x MS2 scRNA
GAAGATCCGGCCTGCAGCCAGTTTTAGAGCTAGAAATAGCAAGTTAAAATAAGGCTAGTCCGTTATCA
ACTTGAAAAAGTGGCACCGAGTCGGTGCGCGCACATGAGGATCACCCATGTGCTTTTTTT

2x MS2 scRNA
GAAGATCCGGCCTGCAGCCAGTTTTAGAGCTAGAAATAGCAAGTTAAAATAAGGCTAGTCCGTTATCA
ACTTGAAAAAGTGGCACCGAGTCGGTGCGGGAGCACATGAGGATCACCCATGTGCCACGAGCGACATG
AGGATCACCCATGTCGCTCGTGTTCCCTTTTTTT

1x MS2 scRNA.b1
GAAGATCCGGCCTGCAGCCAGTTTTAGAGCTAGAAATAGCAAGTTAAAATAAGGCTAGTCCGTTATCA
ACTTGAAAAAGTGCGCACATGAGGATCACCCATGTGCTTTTTTT

1x MS2 scRNA.b2
GAAGATCCGGCCTGCAGCCAGTTTTAGAGCTAGAAATAGCAAGTTAAAATAAGGCTAGTCCGTTATCA
ACTTGAAAAAGTGGCACATGAGGATCACCCATGTGCTTTTTTT

1x PP7 scRNA.b1
GAAGATCCGGCCTGCAGCCAGTTTTAGAGCTAGAAATAGCAAGTTAAAATAAGGCTAGTCCGTTATCA
ACTTGAAAAAGTAACATAAGGAGTTTATATGGAAACCCTTATGTTTTTTT

Annotations:
20 base target site (W108), 1x MS2, tracr RNA terminator, 1x PP7

Candidate activator sequences

Sequences shown below are those candidate activators that gave detectable GFP expression in initial experiments. RNA binding proteins MCP (MS2 coat protein) or PCP (PP7 coat protein) were fused to the N-termini of candidate activator proteins, except for the RpoA αNTD. RpoA has a native flexible linker connecting its N and C terminal domains[17], so we cloned the αNTD with its flexible linker and fused this sequence to the N-terminus of MCP. The MCP and PCP proteins are modified versions ($MCP_{\Delta FG,\ V29I}$ and $PCP_{\Delta FG}$) to prevent oligomerization and improve RNA binding activity[4].

> MCP-(2aa)-SoxS (wtSoxS with 2aa linker used in Fig. 2)

MGPASNFTQFVLVDNGGTGDVTVAPSNFANGIAEWISSNSRSQAYKVTCSVRQSSAQNRKYTIKVEVPKGAWRSYLN
MELTIPIFATNSDCELIVKAMQGLLKDGNPIPSAIAANSGIYGSMSHQKIIQDLIAWIDEHIDQPLNIDVVAKKSGY
SKWYLQRMFRTVTHQTLGDYIRQRRLLLAAVELRTTERPIFDIAMDLGYVSQQTFSRVFRRQFDRTPSDYRHRL

> MCP-(5aa)-$SoxS_{R93A}$ (fully optimized version, Fig. 3 and subsequent)

MGPASNFTQFVLVDNGGTGDVTVAPSNFANGIAEWISSNSRSQAYKVTCSVRQSSAQNRKYTIKVEVPKGAWRSYLN
MELTIPIFATNSDCELIVKAMQGLLKDGNPIPSAIAANSGIYGGGGSMSHQKIIQDLIAWIDEHIDQPLNIDVVAKK
SGYSKWYLQRMFRTVTHQTLGDYIRQRRLLLAAVELRTTERPIFDIAMDLGYVSQQTFSRVFARQFDRTPSDYRHRL

> MCP-(5aa)-TetD (optimized linker for Fig. 5; 2aa linker used in Fig. 2)

MGPASNFTQFVLVDNGGTGDVTVAPSNFANGIAEWISSNSRSQAYKVTCSVRQSSAQNRKYTIKVEVPKGAWRSYLN
MELTIPIFATNSDCELIVKAMQGLLKDGNPIPSAIAANSGIYGGGGSMYIEQHSRYQNKANNIQLRYDDKQFHTTVI
KDVLLWIEHNLDQSLLLDDVANKAGYTKWYFQRLFKKVTGVTLASYIRARRLTKAAVELRLTKKTILEIALKYQFDS
QQSFTRRFKYIFKVTPSYYRRNKLWELEAMH

> MCP-λcII

MGPASNFTQFVLVDNGGTGDVTVAPSNFANGIAEWISSNSRSQAYKVTCSVRQSSAQNRKYTIKVEVPKGAWRSYLN
MELTIPIFATNSDCELIVKAMQGLLKDGNPIPSAIAANSGIYGSMVRANKRNEALRIESALLNKIAMLGTEKTAEAV
GVDKSQISRWKRDWIPKFSMLLAVLEWGVVDDDMARLARQVAAILTNKKRPAATERSEQIQMEF

> MCP-RpoZ

MGPASNFTQFVLVDNGGTGDVTVAPSNFANGIAEWISSNSRSQAYKVTCSVRQSSAQNRKYTIKVEVPKGAWRSYLN
MELTIPIFATNSDCELIVKAMQGLLKDGNPIPSAIAANSGIYGSARVTVQDAVEKIGNRFDLVLVAARRARQMQVGG
KDPLVPEENDKTTVIALREIEEGLINNQILDVRERQEQQEQEAAELQAVTAIAEGRR

> αNTD-MCP

MQGSVTEFLKPRLVDIEQVSSTHAKVTLEPLERGFGHTLGNALRRILLSSMPGCAVTEVEIDGVLHEYSTKEGVQED
ILEILLNLKGLAVRVQGKDEVILTLNKSGIGPVTAADITHDGDVEIVKPQHVICHLTDENASISMRIKVQRGRGYVP
ASTRIHSEEDERPIGRLLVDACYSPVERIAYNVEAARVEQRTDLDKLVIEMETNGTIDPEEAIRRAATILAEQLEAF
VDLRDVRQPEVKEEKPEASNFTQFVLVDNGGTGDVTVAPSNFANGIAEWISSNSRSQAYKVTCSVRQSSAQNRKYTI
KVEVPKGAWRSYLNMELTIPIFATNSDCELIVKAMQGLLKDGNPIPSAIAANSGIY

> MCP-AsiA

MGPASNFTQFVLVDNGGTGDVTVAPSNFANGIAEWISSNSRSQAYKVTCSVRQSSAQNRKYTIKVEVPKGAWRSYLN
MELTIPIFATNSDCELIVKAMQGLLKDGNPIPSAIAANSGIYGSMNKNIDTVREIITVASILIKFSREDIVENRANF
IAFLNEIGVTHEGRKLNQNSFRKIVSELTQEDKKTLIDEFNEGFEGVYRYLEMYTNK

> PCP-(5aa)-$SoxS_{R93A}$

MGPSKTIVLSVGEATRTLTEIQSTADRQIFEEKVGPLVGRLRLTASLRQNGAKTAYRVNLKLDQADVVDSGLPKVRY
TQVWSHDVTIVANSTEASRKSLYDLTKSLVATSQVEDLVVNLVPLGRGGGGSMSHQKIIQDLIAWIDEHIDQPLNID
VVAKKSGYSKWYLQRMFRTVTHQTLGDYIRQRRLLLAAVELRTTERPIFDIAMDLGYVSQQTFSRVFARQFDRTPSD
YRHRL

Fluorescent Protein Reporter Sequences

GFP Reporter (Fig. 2, 3, & 5)
This construct reports on gene activation using a GFP reporter driven by a weak promoter. The promoter was constructed following the design by Bikard et al., 2013[1], driving expression of superfolder GFP (sfGFP)[18]. BBa sequences are from the Repository of Standard Biological Parts (http://parts.igem.org).
W108 target site, BBa_J23117 promoter, Bujard RBS, sfGFP, BBa_B0015 terminator

```
GCATGCCCGATCAACGTCTCATTTTCGCCAGATATCAAGCAGAGGAGCAAAAGCTCATTTCTGAAGAGGACTTGTTG
CGGAAACGACGAGAACAGTTGAAACACAAACTTGAACAGCTACGGAACTCTTGTGCGTAAGGAAAAGTAAGGAAAAC
GATTCCTTCTAACAGAAATGTCCTGAGCAATCACCTATGAACTGTCGACTCGAGCCTCTATGGATTATCACCTTGGC
TGCAGGCCGGATCTTCCACAACACGCACGGTGTTACATTAGGCATACCGGTCttgacagctagctcagtcctaggga
ttgtgctagcGAATTCATTAAAGAGGAGAAAGGTACCATGAGCAAAGGAGAAGAACTTTTCACTGGAGTTGTCCCAA
TTCTTGTTGAATTAGATGGTGATGTTAATGGGCACAAATTTTCTGTCCGTGGAGAGGGTGAAGGTGATGCTACAAAC
GGAAAACTCACCCTTAAATTTATTTGCACTACTGGAAAACTACCTGTTCCGTGGCCAACACTTGTCACTACTCTGAC
CTATGGTGTTCAATGCTTTTCCCGTTATCCGGATCACATGAAACGGCATGACTTTTTCAAGAGTGCCATGCCCGAAG
GTTATGTACAGGAACGCACTATATCTTTCAAAGATGACGGGACCTACAAGACGCGTGCTGAAGTCAAGTTTGAAGGT
GATACCCTTGTTAATCGTATCGAGTTAAAGGGTATTGATTTTAAAGAAGATGGAAACATTCTTGGACACAAACTCGA
GTACAACTTTAACTCACACAATGTATACATCACGGCAGACAAACAAAAGAATGGAATCAAAGCTAACTTCAAAATTC
GCCACAACGTTGAAGATGGTTCCGTTCAACTAGCAGACCATTATCAACAAAATACTCCAATTGGCGATGGCCCTGTC
CTTTTACCAGACAACCATTACCTGTCGACACAATCTGTCCTTTCGAAAGATCCCAACGAAAAGCGTGACCACATGGT
CCTTCTTGAGTTTGTAACTGCTGCTGGGATTACACATGGCATGGATGAGCTCTACAAAtaaggatccaaactcgagt
aaggatctccaggcatcaaataaaacgaaaggctcagtcgaaagactgggcctttcgttttatctgttgtttgtcgg
tgaacgctctctactagagtcacactggctcaccttcgggtgggcctttctgcgtttata
```

RFP Reporter (Fig. 5)
This construct reports on gene silencing using an RFP reporter driven by a strong promoter. The reporter was constructed following the design from Qi et al., 2013[1,13]. The RFP R2 gRNA target site is underlined.
BBa_J23119 promoter, Bujard RBS, mRFP1, BBa_B0015 terminator

```
GCCCTCTAGAGGTGCAAAACCTTTCGCGGTATGGCATGATAGCGCCCGGAAGAGAGTCAATTCAGGGTGGTGAATtt
gacagctagctcagtcctaggtataatagatctGAATTCATTAAAGAGGAGAAAGGTACCATGGCGAGTAGCGAAGA
CGTTATCAAAGAGTTCATGCGTTTCAAAGTTCGTATGGAAGGTTCCGTTAACGGTCACGAGTTCGAAATCGAAGGTG
AAGGTGAAGGTCGTCCGTACGAAGGTACCCAGACCGCTAAACTGAAAGTTACCAAAGGTGGTCCGCTGCCGTTCGCT
TGGGACATCCTGTCCCCGCAGTTCCAGTACGGTTCCAAAGCTTACGTTAAACACCCGGCTGACATCCCGGACTACCT
GAAACTGTCCTTCCCGGAAGGTTTCAAATGGGAACGTGTTATGAACTTCGAAGACGGTGGTGTTGTTACCGTTACCC
AGGACTCCTCCCTGCAAGACGGTGAGTTCATCTACAAAGTTAAACTGCGTGGTACCAACTTCCCGTCCGACGGTCCG
GTTATGCAGAAAAAAACCATGGGTTGGGAAGCTTCCACCGAACGTATGTACCCGGAAGACGGTGCTCTGAAAGGTGA
AATCAAAATGCGTCTGAAACTGAAAGACGGTGGTCACTACGACGCTGAAGTTAAAACCACCTACATGGCTAAAAAAC
CGGTTCAGCTGCCGGGTGCTTACAAAACCGACATCAAACTGGACATCACCTCCCACAACGAAGACTACACCATCGTT
GAACAGTACGAACGTGCTGAAGGTCGTCACTCCACCGGTGCTTAAggatccaaactcgagtaaggatctccaggcat
caaataaaacgaaaggctcagtcgaaagactgggcctttcgttttatctgttgtttgtcggtgaacgctctctacta
gagtcacactggctcaccttcgggtgggcctttctgcgtttata
```

J1 RFP reporter (Fig. 4)
This construct reports on gene activation using an RFP reporter driven by a weak promoter. The J1 upstream region includes PAM sites on both strands every 10 bases to systematically map out the relationship between gRNA target site position and gene activation.
J1 upstream region, BBa_J23117 promoter, Bujard RBS, mRFP1, BBa_B0015 terminator

actcttcctttttcaatattattgaagcatttatcagggttattgtctcatgagcggatacatatttgaatgtattt
agaaaaataaacaaataggggttccgcgcacatttccccgaaaagtgccacctgtggcaattccgacgtcGCCTACG
GTATCCACCGGAGACCTATGGCAGCCTCCGGCCGCCATAGGACACCTTTGGTTGCCAAGGGTGACCTATGGTGACCA
TGGGCCACCACGGGCGACCTCAGGTATCCTGCGGTGTCCTGCGGTTACCAAAGGCGTCCTTTGGGTTCCACCGGATA
CCTCCGGACttgacagctagctcagtcctagggattgtgctagcGAATTCATTAAAGAGGAGAAAGGTACCATGGCG
AGTAGCGAAGACGTTATCAAAGAGTTCATGCGTTTCAAAGTTCGTATGGAAGGTTCCGTTAACGGTCACGAGTTCGA
AATCGAAGGTGAAGGTGAAGGTCGTCCGTACGAAGGTACCCAGACCGCTAAACTGAAAGTTACCAAAGGTGGTCCGC
TGCCGTTCGCTTGGGACATCCTGTCCCCGCAGTTCCAGTACGGTTCCAAAGCTTACGTTAAACACCCGGCTGACATC
CCGGACTACCTGAAACTGTCCTTCCCGGAAGGTTTCAAATGGGAACGTGTTATGAACTTCGAAGACGGTGGTGTTGT
TACCGTTACCCAGGACTCCTCCCTGCAAGACGGTGAGTTCATCTACAAAGTTAAACTGCGTGGTACCAACTTCCCGT
CCGACGGTCCGGTTATGCAGAAAAAAACCATGGGTTGGGAAGCTTCCACCGAACGTATGTACCCGGAAGACGGTGCT
CTGAAAGGTGAAATCAAAATGCGTCTGAAACTGAAAGACGGTGGTCACTACGACGCTGAAGTTAAAACCACCTACAT
GGCTAAAAAACCGGTTCAGCTGCCGGGTGCTTACAAAACCGACATCAAACTGGACATCACCTCCCACAACGAAGACT
ACACCATCGTTGAACAGTACGAACGTGCTGAAGGTCGTCACTCCACCGGTGCTTAAggatccaaactcgagtaagga
tctccaggcatcaaataaaacgaaaggctcagtcgaaagactgggcctttcgttttatctgttgtttgtcggtgaac
gctctctactagagtcacactggctcaccttcgggtgggcctttctgcgtttata

LacZ reporter genes for activity assays at endogenous SoxS target genes

zwf-LacZ reporter

This construct reports on activation of the endogenous Class I SoxS target gene zwf using a zwf-LacZ operon fusion constructed following a previously described design[19]. The construct includes 140 bases upstream of the TSS, the 5' UTR, and coding sequence corresponding to zwf amino acids 1-163 fused in frame to LacZ. The TSS and the soxbox were experimentally identified previously[19-21]. BBa sequences are from the Repository of Standard Biological Parts (http://parts.igem.org).

zwf promoter, zwf_{1-161}, LacZ, BBa_B0015 terminator. The soxbox is underlined.

```
GATCAGTGTCAGATTTTTACCCAATGGAAAACGATGATTTTTTTATCAGTTTTGCCGCACTTTGCGCGCTTTTCCCGTAATCGCAC
GGGTGGATAAGCGTTTACAGTTTTCGCAAGCTCGTAAAAGCAGTACAGTGCACCGTAAGAAAATTACAAGTATACCCTGGCTTAAG
TACCGGGTTAGTTAACTTAAGGAGAATGACATGGCGGTAACGCAAACAGCCCAGGCCTGTGACCTGGTCATTTTCGGCGCGAAAGG
CGACCTTGCGCGTCGTAAATTGCTGCCTTCCCTGTATCAACTGGAAAAAGCCGGTCAGCTCAACCCGGACACCCGGATTATCGGCG
TAGGGCGTGCTGACTGGGATAAAGCGGCATATACCAAAGTTGTCCGCGAGGCGCTCGAAACTTTCATGAAAGAAACCATTGATGAA
GGTTTATGGGACACCCTGAGTGCACGTCTGGATTTTTGTAATCTCGATGTCAATGACACTGCTGCATTCAGCCGTCTCGGCGCGAT
GCTGGATCAAAAAAATCGTATCACCATTAACTACTTTGCCATGCCGCCCAGCACTTTTGGCGCAATTTGCAAAGGGCTTGGCGAGG
CAAAACTGAATGCTAAACCGGCACGCGTAGTCATGGAGAAACCGCTGGGGACGTCGCTGGCGACCTCGCAGGAAATCAATGACATG
ACCATGATTACGGATTCACTGGCCGTCGTTTTACAACGTCGTGACTGGGAAAACCCTGGCGTTACCCAACTTAATCGCCTTGCAGC
ACATCCCCCTTTCGCCAGCTGGCGTAATAGCGAAGAGGCCCGCACCGATCGCCCTTCCCAACAGTTGCGCAGCCTGAATGGCGAAT
GGCGCTTTGCCTGGTTTCCGGCACCAGAAGCGGTGCCGGAAAGCTGGCTGGAGTGCGATCTTCCTGAGGCCGATACTGTCGTCGTC
CCCTCAAACTGGCAGATGCACGGTTACGATGCGCCCATCTACACCAACGTGACCTATCCCATTACGGTCAATCCGCCGTTTGTTCC
CACGGAGAATCCGACGGGTTGTTACTCGCTCACATTTAATGTTGATGAAAGCTGGCTACAGGAAGGCCAGACGCGAATTATTTTTG
ATGGCGTTAACTCGGCGTTTCATCTGTGGTGCAACGGGCGCTGGGTCGGTTACGGCCAGGACAGTCGTTTGCCGTCTGAATTTGAC
CTGAGCGCATTTTTACGCGCCGGAGAAAACCGCCTCGCGGTGATGGTGCTGCGCTGGAGTGACGGCAGTTATCTGGAAGATCAGGA
TATGTGGCGGATGAGCGGCATTTTCCGTGACGTCTCGTTGCTGCATAAACCGACTACACAAATCAGCGATTTCCATGTTGCCACTC
GCTTTAATGATGATTTCAGCCGCGCTGTACTGGAGGCTGAAGTTCAGATGTGCGGCGAGTTGCGTGACTACCTACGGGTAACAGTT
TCTTTATGGCAGGGTGAAACGCAGGTCGCCAGCGGCACCGCGCCTTTCGGCGGTGAAATTATCGATGAGCGTGGTGGTTATGCCGA
TCGCGTCACACTACGTCTGAACGTCGAAAACCCGAAACTGTGGAGCGCCGAAATCCCGAATCTCTATCGTGCGGTGGTTGAACTGC
ACACCGCCGACGGCACGCTGATTGAAGCAGAAGCCTGCGATGTCGGTTTCCGCGAGGTGCGGATTGAAAATGGTCTGCTGCTGCTG
AACGGCAAGCCGTTGCTGATTCGAGGCGTTAACCGTCACGAGCATCATCCTCTGCATGGTCAGGTCATGGATGAGCAGACGATGGT
GCAGGATATCCTGCTGATGAAGCAGAACAACTTTAACGCCGTGCGCTGTTCGCATTATCCGAACCATCCGCTGTGGTACACGCTGT
GCGACCGCTACGGCCTGTATGTGGTGGATGAAGCCAATATTGAAACCCACGGCATGGTGCCAATGAATCGTCTGACCGATGATCCG
CGCTGGCTACCGGCGATGAGCGAACGCGTAACGCGAATGGTGCAGCGCGATCGTAATCACCCGAGTGTGATCATCTGGTCGCTGGG
GAATGAATCAGGCCACGGCGCTAATCACGACGCGCTGTATCGCTGGATCAAATCTGTCGATCCTTCCCGCCCGGTGCAGTATGAAG
GCGGCGGAGCCGACACCACGGCCACCGATATTATTTGCCCGATGTACGCGCGCGTGGATGAAGACCAGCCCTTCCCGGCTGTGCCG
AAATGGTCCATCAAAAAATGGCTTTCGCTACCTGGAGAGACGCGCCCGCTGATCCTTTGCGAATACGCCCACGCGATGGGTAACAG
TCTTGGCGGTTTCGCTAAATACTGGCAGGCGTTTCGTCAGTATCCCCGTTTACAGGGCGGCTTCGTCTGGGACTGGGTGGATCAGT
CGCTGATTAAATATGATGAAAACGGCAACCCGTGGTCGGCTTACGGCGGTGATTTTGGCGATACGCCGAACGATCGCCAGTTCTGT
ATGAACGGTCTGGTCTTTGCCGACCGCACGCCGCATCCAGCGCTGACGGAAGCAAAACACCAGCAGCAGTTTTTCCAGTTCCGTTT
ATCCGGGCAAACCATCGAAGTGACCAGCGAATACCTGTTCCGTCATAGCGATAACGAGCTCCTGCACTGGATGGTGGCGCTGGATG
GTAAGCCGCTGGCAAGCGGTGAAGTGCCTCTGGATGTCGCTCCACAAGGTAAACAGTTGATTGAACTGCCTGAACTACCGCAGCCG
GAGAGCGCCGGGCAACTCTGGCTCACAGTACGCGTAGTGCAACCGAACGCGACCGCATGGTCAGAAGCCGGGCACATCAGCGCCTG
GCAGCAGTGGCGTCTGGCGGAAAACCTCAGTGTGACGCTCCCCGCCGCGTCCCACGCCATCCCGCATCTGACCACCAGCGAAATGG
ATTTTTGCATCGAGCTGGGTAATAAGCGTTGGCAATTTAACCGCCAGTCAGGCTTTCTTTCACAGATGTGGATTGGCGATAAAAAA
CAACTGCTGACGCCGCTGCGCGATCAGTTCACCCGTGCACCGCTGGATAACGACATTGGCGTAAGTGAAGCGACCCGCATTGACCC
TAACGCCTGGGTCGAACGCTGGAAGGCGGCGGGCCATTACCAGGCCGAAGCAGCGTTGTTGCAGTGCACGGCAGATACACTTGCTG
ATGCGGTGCTGATTACGACCGCTCACGCGTGGCAGCATCAGGGGAAAACCTTATTTATCAGCCGGAAAACCTACCGGATTGATGGT
AGTGGTCAAATGGCGATTACCGTTGATGTTGAAGTGGCGAGCGATACACCGCATCCGGCGCGGATTGGCCTGAACTGCCAGCTGGC
GCAGGTAGCAGAGCGGGTAAACTGGCTCGGATTAGGGCCGCAAGAAAACTATCCCGACCGCCTTACTGCCGCCTGTTTTGACCGCT
GGGATCTGCCATTGTCAGACATGTATACCCCGTACGTCTTCCCGAGCGAAAACGGTCTGCGCTGCGGGACGCGCGAATTGAATTAT
GGCCCACACCAGTGGCGCGGCGACTTCCAGTTCAACATCAGCCGCTACAGTCAACAGCAACTGATGGAAACCAGCCATCGCCATCT
GCTGCACGCGGAAGAAGGCACATGGCTGAATATCGACGGTTTCCATATGGGGATTGGTGGCGACGACTCCTGGAGCCCGTCAGTAT
CGGCGGAATTCCAGCTGAGCGCCGGTCGCTACCATTACCAGTTGGTCTGGTGTCAAAAATAAggatccaaactcgagtaaggatct
ccaggcatcaaataaaacgaaaggctcagtcgaaagactgggcctttcgttttatctgttgtttgtcggtgaacgctctctactag
agtcacactggctcaccttcgggtgggcctttctgcgtttata
```

fumC-LacZ reporter

This construct reports on activation of the endogenous Class II SoxS target gene fumC using a fumC-LacZ operon fusion. The construct includes 46 bases upstream of the TSS, the 5' UTR, and coding sequence corresponding to fumC amino acids 1-56 fused in frame to LacZ. This construct is similar but not identical to a previously described fumC-LacZ reporter gene[22]. The TSS and the soxbox were experimentally identified previously[21,23]. BBa sequences are from the Repository of Standard Biological Parts (http://parts.igem.org).

fumC promoter, $fumC_{1-56}$, LacZ, BBa_B0015 terminator. The soxbox is underlined.

ATGGCACGAAAGACCAAACATTTGTTATCAAATGGTAAATAATAAGTGAGCTAAAAGTTGCTTAACGAAAGCAAAACAGAAAGAAA
AAATTAATCAGGTGAGGAGCAGGTCATGAATACAGTACGCAGCGAAAAAGATTCGATGGGGGCGATTGATGTCCCGGCAGATAAGC
TGTGGGGCGCACAAACTCAACGCTCGCTGGAGCATTTCCGCATTTCGACGGAGAAAATGCCCACCTCACTGATTCATGCGCTGGCG
CTAACCAAGCGTGCAGCGGCCATGACCATGATTACGGATTCACTGGCCGTCGTTTTACAACGTCGTGACTGGGAAAACCCTGGCGT
TACCCAACTTAATCGCCTTGCAGCACATCCCCCTTTCGCCAGCTGGCGTAATAGCGAAGAGGCCCGCACCGATCGCCCTTCCCAAC
AGTTGCGCAGCCTGAATGGCGAATGGCGCTTTGCCTGGTTTCCGGCACCAGAAGCGGTGCCGGAAAGCTGGCTGGAGTGCGATCTT
CCTGAGGCCGATACTGTCGTCGTCCCCTCAAACTGGCAGATGCACGGTTACGATGCGCCCATCTACACCAACGTGACCTATCCCAT
TACGGTCAATCCGCCGTTTGTTCCCACGGAGAATCCGACGGGTTGTTACTCGCTCACATTTAATGTTGATGAAAGCTGGCTACAGG
AAGGCCAGACGCGAATTATTTTTGATGGCGTTAACTCGGCGTTTCATCTGTGGTGCAACGGGCGCTGGGTCGGTTACGGCCAGGAC
AGTCGTTTGCCGTCTGAATTTGACCTGAGCGCATTTTTACGCGCCGGAGAAAACCGCCTCGCGGTGATGGTGCTGCGCTGGAGTGA
CGGCAGTTATCTGGAAGATCAGGATATGTGGCGGATGAGCGGCATTTTCCGTGACGTCTCGTTGCTGCATAAACCGACTACACAAA
TCAGCGATTTCCATGTTGCCACTCGCTTTAATGATGATTTCAGCCGCGCTGTACTGGAGGCTGAAGTTCAGATGTGCGGCGAGTTG
CGTGACTACCTACGGGTAACAGTTTCTTTATGGCAGGGTGAAACGCAGGTCGCCAGCGGCACCGCGCCTTTCGGCGGTGAAATTAT
CGATGAGCGTGGTGGTTATGCCGATCGCGTCACACTACGTCTGAACGTCGAAAACCCGAAACTGTGGAGCGCCGAAATCCCGAATC
TCTATCGTGCGGTGGTTGAACTGCACACCGCCGACGGCACGCTGATTGAAGCAGAAGCCTGCGATGTCGGTTTCCGCGAGGTGCGG
ATTGAAAATGGTCTGCTGCTGCTGAACGGCAAGCCGTTGCTGATTCGAGGCGTTAACCGTCACGAGCATCATCCTCTGCATGGTCA
GGTCATGGATGAGCAGACGATGGTGCAGGATATCCTGCTGATGAAGCAGAACAACTTTAACGCCGTGCGCTGTTCGCATTATCCGA
ACCATCCGCTGTGGTACACGCTGTGCGACCGCTACGGCCTGTATGTGGTGGATGAAGCCAATATTGAAACCCACGGCATGGTGCCA
ATGAATCGTCTGACCGATGATCCGCGCTGGCTACCGGCGATGAGCGAACGCGTAACGCGAATGGTGCAGCGCGATCGTAATCACCC
GAGTGTGATCATCTGGTCGCTGGGGAATGAATCAGGCCACGGCGCTAATCACGACGCGCTGTATCGCTGGATCAAATCTGTCGATC
CTTCCCGCCCGGTGCAGTATGAAGGCGGCGGAGCCGACACCACGGCCACCGATATTATTTGCCCGATGTACGCGCGCGTGGATGAA
GACCAGCCCTTCCCGGCTGTGCCGAAATGGTCCATCAAAAAATGGCTTTCGCTACCTGGAGAGACGCGCCCGCTGATCCTTTGCGA
ATACGCCCACGCGATGGGTAACAGTCTTGGCGGTTTCGCTAAATACTGGCAGGCGTTTCGTCAGTATCCCCGTTTACAGGGCGGCT
TCGTCTGGGACTGGGTGGATCAGTCGCTGATTAAATATGATGAAAACGGCAACCCGTGGTCGGCTTACGGCGGTGATTTTGGCGAT
ACGCCGAACGATCGCCAGTTCTGTATGAACGGTCTGGTCTTTGCCGACCGCACGCCGCATCCAGCGCTGACGGAAGCAAAACACCA
GCAGCAGTTTTTCCAGTTCCGTTTATCCGGGCAAACCATCGAAGTGACCAGCGAATACCTGTTCCGTCATAGCGATAACGAGCTCC
TGCACTGGATGGTGGCGCTGGATGGTAAGCCGCTGGCAAGCGGTGAAGTGCCTCTGGATGTCGCTCCACAAGGTAAACAGTTGATT
GAACTGCCTGAACTACCGCAGCCGGAGAGCGCCGGGCAACTCTGGCTCACAGTACGCGTAGTGCAACCGAACGCGACCGCATGGTC
AGAAGCCGGGCACATCAGCGCCTGGCAGCAGTGGCGTCTGGCGGAAAACCTCAGTGTGACGCTCCCCGCCGCGTCCCACGCCATCC
CGCATCTGACCACCAGCGAAATGGATTTTTGCATCGAGCTGGGTAATAAGCGTTGGCAATTTAACCGCCAGTCAGGCTTTCTTTCA
CAGATGTGGATTGGCGATAAAAAACAACTGCTGACGCCGCTGCGCGATCAGTTCACCCGTGCACCGCTGGATAACGACATTGGCGT
AAGTGAAGCGACCCGCATTGACCCTAACGCCTGGGTCGAACGCTGGAAGGCGGCGGGCCATTACCAGGCCGAAGCAGCGTTGTTGC
AGTGCACGGCAGATACACTTGCTGATGCGGTGCTGATTACGACCGCTCACGCGTGGCAGCATCAGGGGAAAACCTTATTTATCAGC
CGGAAAACCTACCGGATTGATGGTAGTGGTCAAATGGCGATTACCGTTGATGTTGAAGTGGCGAGCGATACACCGCATCCGGCGCG
GATTGGCCTGAACTGCCAGCTGGCGCAGGTAGCAGAGCGGGTAAACTGGCTCGGATTAGGGCCGCAAGAAAACTATCCCGACCGCC
TTACTGCCGCCTGTTTTGACCGCTGGGATCTGCCATTGTCAGACATGTATACCCCGTACGTCTTCCCGAGCGAAAACGGTCTGCGC
TGCGGGACGCGCGAATTGAATTATGGCCCACACCAGTGGCGCGGCGACTTCCAGTTCAACATCAGCCGCTACAGTCAACAGCAACT
GATGGAAACCAGCCATCGCCATCTGCTGCACGCGGAAGAAGGCACATGGCTGAATATCGACGGTTTCCATATGGGGATTGGTGGCG
ACGACTCCTGGAGCCCGTCAGTATCGGCGGAATTCCAGCTGAGCGCCGGTCGCTACCATTACCAGTTGGTCTGGTGTCAAAAATAA
ggatccaaactcgagtaaggatctccaggcatcaaataaaacgaaaggctcagtcgaaagactgggcctttcgttttatctgttgt
ttgtcggtgaacgctctctactagagtcacactggctcaccttcgggtgggcctttctgcgtttata

Ethanol Production Pathway Cassette (pCD355)

This construct includes the *Z. mobilis pdc-adhB* gene cluster driven by a weak promoter. In Fig. 6, the CRISPRa complex targets the J106 site upstream of the promoter. J106 is the site with maximal activity in reporter assays (Fig. 4B).

J1 upstream region (J106 site underlined), BBa_J23117 promoter, Bujard RBS, Zm.pdc, Zm.adhB, BBa_B0015 terminator

gcgacacggaaatgttgaatactcatactcttcctttttcaatattattgaagcatttatcagggttattgtctcat
gagcggatacatatttgaatgtatttagaaaaataaacaaataggggttccgcgcacatttccccgaaaagtgccac
ctgACGTCGCGGCCGCCTACGGTATCCACCGGAGACCTATGGCAGCCTCCGGCCGCCATAGGACACCTTTGGTTGCC
AAGGGTGACCTATGGTGACCATGGGCCACCACGGGCGACCTCAGGTATCCTGCGGTGTCCTGCGGTTACCAAAGGCG
TCCTTTGGGTTCCACCGGATACCTCCGGACttgacagctagctcagtcctagggattgtgctagcGAATTCATTAAA
GAGGAGAAAGGTACCatgagttatactgtcggtacctatttagcggagcggcttgtccagattggtctcaagcatca
cttcgcagtcgcgggcgactacaacctcgtccttcttgacaacctgcttttgaacaaaaacatggagcaggtttatt
gctgtaacgaactgaactgcggtttcagtgcagaaggttatgctcgtgccaaaggcgcagcagcagccgtcgttacc
tacagcgtcggtgcgctttccgcatttgatgctatcggtggcgcctatgcagaaaaccttccggttatcctgatctc
cggtgctccgaacaacaatgaccacgctgctggtcacgtgttgcatcacgctcttggcaaaaccgactatcactatc
agttggaaatggccaagaacatcacggccgccgctgaagcgatttataccccggaagaagctccggctaaaatcgat
cacgtgattaaaactgctcttcgtgagaagaagccggtttatctcgaaatcgcttgcaacattgcttccatgccctg
cgccgctcctggaccggcaagcgcattgttcaatgacgaagccagcgacgaagcttctttgaatgcagcggttgaag
aaaccctgaaattcatcgccnaccgcgacaaagttgccgtcctcgtcggcagcaagctgcgcgcagctggtgctgaa
gaagctgctgtcaaatttgctgatgctcttggtggcgcagttgctaccatggctgctgcaaaaagcttcttcccaga
agaaaacccgcattacatcggtacctcatggggtgaagtcagctatccgggcgttgaaaagacgatgaaagaagccg
atgcggttatcgctctggctcctgtctttaacgactactccaccactggttggacggatattcctgatcctaagaaa
ctggttctcgctgaaccgcgttctgtcgtcgttaacggcattcgcttccccagcgtccatctgaaagactatctgac
ccgtttggctcagaaagtttccaagaaaaccggtgctttggacttcttcaaatccctcaatgcaggtgaactgaaga
aagccgctccggctgatccgagtgctccgttggtcaacgcagaaatcgcccgtcaggtcgaagctcttctgaccccg
aacacgacggttattgctgaaaccggtgactcttggttcaatgctcagcgcatgaagctcccgaacggtgctcgcgt
tgaatatgaaatgcagtggggtcacattggttggtccgttcctgccgccttcggttatgccgtcggtgctccggaac
gtcgcaacatcctcatggttggtgatggttccttccagctgacggctcaggaagtcgctcagatggttcgcctgaaa
ctgccggttatcatcttcttgatcaataactatggttacaccatcgaagttatgatccatgatggtccgtacaacaa
catcaagaactgggattatgccggtctgatggaagtgttcaacggtaacggtggttatgacagcggtgctggtaaag
gcctgaaggctaaaaccggtggcgaactggcagaagctatcaaggttgctctggcaaacaccgacggcccaaccctg
atcgaatgcttcatcggtcgtgaagactgcactgaagaattggtcaaatggggtaagcgcgttgctgccgccaacag
ccgtaagcctgttaacaagctcctctaacaattcaaaaGATCTAAAGAGGAGAAATCTAGAatggcttcttcaactt
tttatattcctttcgtcaacgaaatgggcgaaggttcgcttgaaaaagcaatcaaggatcttaacggcagcggcttt
aaaaatgcgctgatcgtttctgatgctttcatgaacaaatccggtgttgtgaagcaggttgctgacctgttgaaagc
acagggtattaattctgctgtttatgatggcgttatgccgaacccgactgttaccgcagttctggaaggccttaaga
tcctgaaggataacaattcagacttcgtcatctccctcggtggtggttctccccatgactgcgccaaagccatcgct
ctggtcgcaaccaatggtggtgaagtcaaagactacgaaggtatcgacaaatctaagaaacctgccctgcctttgat
gtcaatcaacacgacggctggtacggcttctgaaatgacgcgtttctgcatcatcactgatgaagtccgtcacgtta
agatggccattgttgaccgtcacgttaccccgatggtttccgtcaacgatcctctgttgatggttggtatgccaaaa
ggcctgaccgccgccaccggtatggatgctctgacccacgcatttgaagcttattcttcaacggcagctactccgat
caccgatgcttgcgctttgaaagcagcttccatgatcgctaagaatctgaagaccgcttgcgacaacggtaaggata
tgccggctcgtgaagctatggcttatgcccaattcctcgctggtatggccttcaacaacgcttcgcttggttatgtc
catgctatggctcaccagttgggcggttactacaacctgccgcatggtgtctgcaacgctgttctgcttccgcatgt
tctggcttataacgcctctgtcgttgctggtcgtctgaaagacgttggtgttgctatgggtctcgatatcgccaatc
tcggtgataaagaaggcgcagaagccaccattcaggctgttcgcgatctggctgcttccattggtattccagcaaac
ctgaccgagctgggtgctaagaaagaagatgtgccgcttcttgctgaccacgctctgaaagatgcttgtgctctgac
caacccgcgtcagggtgatcagaaagaagttgaagaactcttcctgagcgctttctaaggatccaaactcgagtaag
gatctCCAGGCATCAAATAAAACGAAAGGCTCAGTCGAAAGACTGGGCCTTTCGTTTTATCTGTTGTTTGTCGGTGA
ACGCTCTCTACTAGAGTCACACTGGCTCACCTTCGGGTGGGCCTTTCTGCGTTTATA

Supplementary References

1. Bikard, D. *et al.* Programmable repression and activation of bacterial gene expression using an engineered CRISPR-Cas system. *Nucleic Acids Res.* **41,** 7429–7437 (2013).
2. Dove, S. L. & Hochschild, A. Conversion of the omega subunit of *Escherichia coli* RNA polymerase into a transcriptional activator or an activation target. *Genes Dev.* **12,** 745–754 (1998).
3. Gregory, B. D., Deighan, P. & Hochschild, A. An artificial activator that contacts a normally occluded surface of the RNA polymerase holoenzyme. *J. Mol. Biol.* **353,** 497–506 (2005).
4. Zalatan, J. G. *et al.* Engineering complex synthetic transcriptional programs with CRISPR RNA scaffolds. *Cell* **160,** 339–350 (2015).
5. Konermann, S. *et al.* Genome-scale transcriptional activation by an engineered CRISPR-Cas9 complex. *Nature* **517,** 583–588 (2015).
6. Ho, Y. S., Mahoney, M. E., Wulff, D. L. & Rosenberg, M. Identification of the DNA binding domain of the phage lambda cII transcriptional activator and the direct correlation of cII protein stability with its oligomeric forms. *Genes Dev.* **2,** 184–195 (1988).
7. Griffith, K. L. & Wolf, R. E. A comprehensive alanine scanning mutagenesis of the *Escherichia coli* transcriptional activator SoxS: identifying amino acids important for DNA binding and transcription activation. *J. Mol. Biol.* **322,** 237–257 (2002).
8. Zhang, X. & Bremer, H. Control of the *Escherichia coli* rrnB P1 promoter strength by ppGpp. *J. Biol. Chem.* **270,** 11181–11189 (1995).
9. Livak, K. J. & Schmittgen, T. D. Analysis of relative gene expression data using real-time quantitative PCR and the $2^{-\Delta\Delta C(T)}$ method. *Methods* **25,** 402–408 (2001).
10. Shah, I. M. & Wolf, R. E. Novel protein-protein interaction between Escherichia coli SoxS and the DNA binding determinant of the RNA polymerase alpha subunit: SoxS functions as a co-sigma factor and redeploys RNA polymerase from UP-element-containing promoters to SoxS-dependent promoters during oxidative stress. *J. Mol. Biol.* **343,** 513–532 (2004).
11. Sievers, F. *et al.* Fast, scalable generation of high-quality protein multiple sequence alignments using Clustal Omega. *Mol. Syst. Biol.* **7,** 539 (2011).
12. Shapiro, H. M. Multiparameter flow cytometry of bacteria: implications for diagnostics and therapeutics. *Cytometry* **43,** 223–226 (2001).
13. Qi, L. S. *et al.* Repurposing CRISPR as an RNA-guided platform for sequence-specific control of gene expression. *Cell* **152,** 1173–1183 (2013).
14. Lee, T. S. *et al.* BglBrick vectors and datasheets: A synthetic biology platform for gene expression. *J. Biol. Eng.* **5,** 12 (2011).
15. Jinek, M. *et al.* A programmable dual-RNA-guided DNA endonuclease in adaptive bacterial immunity. *Science* **337,** 816–821 (2012).
16. Nishimasu, H. *et al.* Crystal structure of Cas9 in complex with guide RNA and target DNA. *Cell* **156,** 935–949 (2014).
17. Blatter, E. E., Ross, W., Tang, H., Gourse, R. L. & Ebright, R. H. Domain organization of RNA polymerase alpha subunit: C-terminal 85 amino acids constitute a domain capable of dimerization and DNA binding. *Cell* **78,** 889–896 (1994).
18. Pédelacq, J.-D., Cabantous, S., Tran, T., Terwilliger, T. C. & Waldo, G. S. Engineering and characterization of a superfolder green fluorescent protein. *Nat. Biotechnol.* **24,** 79–88

(2006).
19. Fawcett, W. P. & Wolf, R. E. Genetic definition of the *Escherichia coli zwf* ‘soxbox,’ the DNA binding site for SoxS-mediated induction of glucose 6-phosphate dehydrogenase in response to superoxide. *J. Bacteriol.* **177,** 1742–1750 (1995).
20. Rowley, D. L. & Wolf, R. E. Molecular characterization of the Escherichia coli K-12 zwf gene encoding glucose 6-phosphate dehydrogenase. *J. Bacteriol.* **173,** 968–977 (1991).
21. Martin, R. G., Gillette, W. K., Rhee, S. & Rosner, J. L. Structural requirements for marbox function in transcriptional activation of mar/sox/rob regulon promoters in Escherichia coli: sequence, orientation and spatial relationship to the core promoter. *Mol. Microbiol.* **34,** 431–441 (1999).
22. Fawcett, W. P. & Wolf, R. E. Purification of a MalE-SoxS fusion protein and identification of the control sites of *Escherichia coli* superoxide-inducible genes. *Mol. Microbiol.* **14,** 669–679 (1994).
23. Taliaferro, L. P., Keen, E. F., Sanchez-Alberola, N. & Wolf, R. E. Transcription activation by Escherichia coli Rob at class II promoters: protein-protein interactions between Rob's N-terminal domain and the σ(70) subunit of RNA polymerase. *J. Mol. Biol.* **419,** 139–157 (2012).
